# Does revascularisation for residual ischaemia in patients with ACS influence prognosis?

**DOI:** 10.1186/1532-429X-18-S1-O59

**Published:** 2016-01-27

**Authors:** Neha Sekhri, Kenneth Fung, Mohammed H Iqbal, Mohammed O Anwar, Daniel A Jones, Anthony Mathur, Andrew Wragg, Adam Timmis

**Affiliations:** 1 Barts Heart Centre, Barts Health NHS Trust, London, UK; 2 Barts and the London School of Medicine and Dentistry, London, UK

## Background

Residual myocardial ischaemia early after acute coronary syndromes (ACS) is commonly regarded as an adverse prognostic sign and an indication for revascularisation. However, the benefits of revascularisation for improving prognosis are not known.

## Methods

Analysis of 598 consecutive patients with ACS treated with coronary stenting, all of whom underwent adenosine stress cardiac magnetic resonance (CMR) perfusion imaging to guide revascularisation decisions. Follow-up data were obtained from hospital electronic health records.

## Results

**Figure 1:**
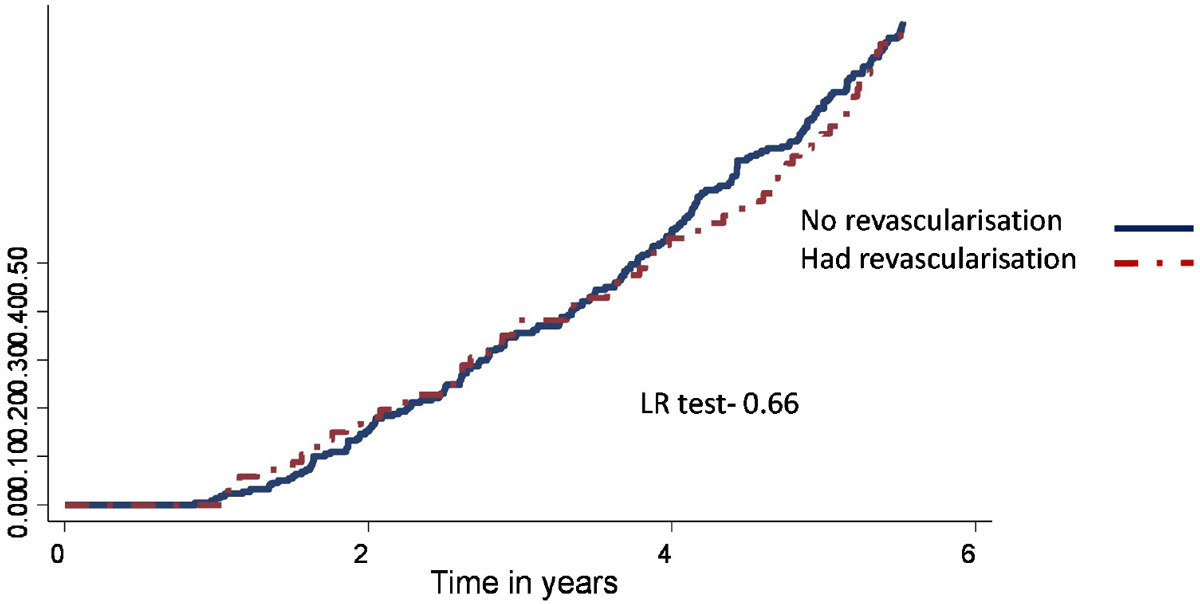
**Probability of all cause mortality in patients with perfusion defect stratified by revascularisation**.

The 598 patients (age 59 ± 12 years, 20% female underwent stress CMR a median of 93 days (IQR: 41, 224 days) after coronary stenting with follow-up for 1.4 years (IQR: 0.6-2.7). Inducible perfusion defects were identified in 294 (49%) patients of whom 18 (6%) died during follow-up compared with 6 (2.0%) patients with no perfusion defects (p = 0.01). Of the 294 patients with perfusion defects, 70 (24%) were revascularised (PCI 55, CABG 27) of whom 5 (7%) died during follow-up compared with 13 (6%) who were not revascularised(p = 0.68). K-M survival analysis confirmed that revascularisation was unassociated with survival benefit, regardless of the severity of ischaemia (Figure 1).

## Conclusions

In our patients with ACS and coronary stenting, inducible ischaemia was associated with increased risk of death during follow-up. Revascularisation did not appear to reduce the risk and should be reserved for improving symptoms in patients on optimal medical therapy.

